# The Development of Emotion Understanding among Five- and Six-Year-Old Left-Behind Children in Rural China

**DOI:** 10.3390/ijerph20053974

**Published:** 2023-02-23

**Authors:** Ruifeng Tan, Huimin Fang, Suiqing Chen

**Affiliations:** School of Education, Guangzhou University, Guangzhou 510006, China

**Keywords:** left-behind children, parental migration, early childhood, emotion understanding

## Abstract

The left-behind children (LBC), separated from their mother/father or parents for a long period of time, have long been discussed as a subject of concern in China. Existing research has concluded that rural children who did not migrate with parents are subject to emotional risks. In the present study, the purpose is to study the impact of parental migration on early emotional understanding. Purposeful sampling was used to recruit 180 children aged five to six years in rural areas of Guangdong province, including LBC and non-left-behind children (NLBC). Their level of emotional understanding (EU) was assessed by the emotional comprehension test (TEC) adapted to the Chinese context. The results showed that, on the three levels (*External*, *Internal*, *Reflective*) of emotional understanding, LBC aged five- to six- years old scored significantly lower than NLBC as counterparts. On the whole, the emotional comprehension ability of preschool LBC was significantly lower than that of NLBC. However, there were no significant differences within LBC nurtured by single parents, grandparents, and other relatives. This study confirmed that parental migration in early childhood considerably impacted rural LBC’s emotional understanding and affectional adjustment, which provided a significant basis for increasing parental care and early childhood companionship in rural areas.

## 1. Introduction

In China, social development is mainly reflected in urbanization, which has brought about labor migration (rural–urban and urban–urban) to meet the needs of urban industrial development and alleviate the pressure of living in a rural family setting [1]. However, children who are left behind at home are perceived to have special needs resulting from China’s big labor movement, and their special situation has attracted the attention of the whole society. One of the main concerns deals with the psychological adaptation of left-behind children (LBC), defined as children who have one or both migrant parents working in cities leaving them with other caregivers at home [2]. Someone argued that parental migration has adverse effects on young children’s mental well-being [3]. Other research indicated that the situation of LBC was so difficult to handle that the impact on children’s well-being and socialization was prone to being ignored or mixed [4]. Emotional interpretation is critical for the social development of young children. The integrity and stability of the children’s family structures may play a large role in this development [5]. In the context of a rural revitalization strategy [6], a considerable amount of research about the status of LBC as the product of social migration has drawn wide attention over the past couple of years. Exploring the level of emotional understanding of Chinese LBC in early childhood would further promote the understanding of this special population and their families.

### 1.1. Emotion Understanding in Early Childhood

Understanding of emotional states refers to the ability to identify, express, and communicate regarding comprehension of one’s own and others’ psychological activities, behaviors, and situations [7,8]. According to existing research, emotional understanding (EU) generally includes a set of skills that enable the understanding of some emotional elements or events (regulation of an experienced emotion, distinction between different emotions experienced, and external expression of what they feel) [9]. This competence mainly involves the understanding of facial expressions, situation-based emotions, desire-based emotions, belief-based emotions, reminder-based emotions, emotional display rules or hiding emotions, mixed emotions, moral emotions, emotional attribution or external cause, and emotional regulation [10,11]. Furthermore, the previous reviews and studies presented three hierarchical levels of emotional understanding, including “*External*” (e.g., recognition, situation, and reminder); “*Internal*” (e.g., belief, desire, and display); and “*Reflective*” (e.g., mixed, regulation, cause, and morality) [12,13,14,15]. In accordance with differential emotions theory and the social processing theory of emotion, early emotional development would change through neural maturation and socialization experiences [16,17]. Moreover, much evidence has examined that children’s understanding of emotions was highly correlated with social competence, as well as being especially linked to children’s prosocial behaviors [18]. This means early emotional understanding may also be discrete or divergent.

Children’s early emotional understanding also could be divided into different stages [19,20]. Almost all the three-year-old children were able to recognize a minimum of four out of five emotions based on facial expression alone [11]. Around the age of five, children appreciated the fact that people have different desires and beliefs, and they even correctly identify external causes and understand the impact of reminders about those emotions [11,21]. Some research has indicated that children aged five began to experience and understand mixed emotions [22]. Therefore, the levels of early competence to understand emotions would best be examined at around the age of five to six years old.

### 1.2. Parental Presence and Child’s Emotional Development

The social-ecological model proposes that children’s development is inextricably linked to the multilevel environment, especially parental care [23]. Parental presence is of great significance to the emotional and mental health of children. According to parent-child attachment theory, parental company was significantly related to children’s emotional functioning, especially their emotional understanding [24,25].

According to the parental acceptance–rejection theory, paternal neglect correlates closely with children’s psychological maladjustment [26]. The absence of parents hindered children’s feelings of parent-child cohesion and psychological needs satisfaction [27]. The impact of parental separation on children’s emotional indifference was not only short-term, but lasted well into adulthood [28]. The longer the early childhood separation, the more negative the effect of parental absence on psychological well-being, like emotional desertification [29,30]. Moreover, parental socialization of emotion was related to children’s emotional comprehension [31]. Therefore, the absence of parents may have an important impact on EU in early childhood.

### 1.3. Left-Behind Children in China

The living standard has increased rapidly in China since the 1980s, followed by large-scale population movements. In the countryside, children and adolescents were left behind at home by their rural-to-urban migrant parents in search of better jobs as a result of the household registration system [32]. In 2018, the LBC population reached 6.97 million [33]. Regarding the duration of the separation, the length of parental absence for LBC was generally more than six months [34]. From the perspective of family system theory [35], the changes of parental migration and family life, due to a trade-off between an increase in family income and a decrease in parental care causes, has seemingly contradictory impacts on LBC’s social and emotional adjustment [36].

Close parent–child emotional bonding can occur only through physical closeness between parents and their children, such as physical contact, caresses, kisses, and clear demonstrations of affection [37]. LBC obviously lacked such opportunities for intimate contact and were easily exposed to all kinds of psycho-social well-being risks [38,39], especially those involving core emotional level [40]. Children’s emotion comprehension was closely correlated to parents and their interactions [41,42]. Particularly, LBC would have more difficulty frequently communicating with their parents. Therefore, they would suffer more emotional crises than NLBC [43,44]. A meta-analytic review showed that most studies focused on the emotional functioning of LBC over the age of seven [45]. In fact, the first five years was the critical period for emotional and social development [1]. Therefore, the effect of early parental separation on a child’s emotional adaptation should receive more attention [46,47,48]. Furthermore, the impact of LBC’s actual caregivers on their EU was not yet clear.

### 1.4. The Current Study

Therefore, the present study sought to analyze the effect of the experience of being left behind and caregiver types on EU in early childhood. We explored whether five- and six-year-old LBC and non-left-behind children (NLBC) differed with regard to the three levels (*External*, *Internal*, *Reflective*) of understanding emotions. This study explored whether there were EU differences for young LBC living with different family members or actual caregivers. In terms of caregivers or guardians, referring to previous research [49,50], three types of LBC’s caregivers were considered: single parent, grandparents, or other relatives. Since living alone or only with siblings is rarely present at the preschool ages, it was not considered. Therefore, it could be predicted that LBC aged five to six years would score lower on the three levels of EU than NLBC.

## 2. Methods

### 2.1. Participants

The participants were 200 young children invited randomly from kindergartens in rural Guangdong province. Excluding 20 participants with invalid and wrong data, the final sample size was 180, among which 90 children whose parents had left them behind at least six months prior and 90 young children who still had both parents living at home. Among the LBC, there were equal numbers of boys and girls, 45 in each group. Moreover, there were 30 LBC in each of the three caregiver types. The inclusion criteria for participants in the study were preschool children aged five and six years, having receptive language and picture processing skills, as well as mental-state and conversational stability [51,52,53]. The study was reviewed approved by the research ethics committee of Guangzhou University (Protocol Number: GZHU 2019019).

### 2.2. Design

To compare the differences in EU between LBC and NLBC, the study used two mixed design of 2 (Gender: Boy or girl) × 2 (Children’s status: Left-behind or non-left-behind). Gender and children’s status were independent variable, and the scores of emotional understanding were dependent variables. The age differences between the two groups were compared and the T-test results showed that the difference was not significant (*t*_(178)_ = 0.28, *p* = 0.56, *Cohen’s d* = 0.76).

### 2.3. Materials and Procedures

In order to exclude the influence of the participants by the age or gender of the protagonists of the picture, this study specially found two children of similar age to the research samples. A seven-point scale questionnaire was produced, and twenty-five kindergarten teachers rated the facial expressions pictures and their naming (1 is very inconsistent, 7 is very consistent), and then picked the facial expression photos with the high score. Therefore, ten pictures of facial expressions (e.g., *happy*, *sad*, *angry*, *fearful and neutral*) of children aged 5 to 6 were successfully selected as basic materials (to create an equal gender endowment). The expressions of real children and similar ages made them easier for participants to understand and identify. Based on the results of the initial examination of the general child, these photos can convey the corresponding emotions.

The emotion comprehension test (TEC) has been widely translated into 25 languages to assess children’s levels of understanding emotions before this study. It has shown good test–retest reliability, as well as concurrent, criterion, and construct validity [10,20]. The standardized Chinese version of the TEC was successfully adapted and administered in Chinese preschool children [12,13]. The test materials consisted of the sample scenarios. Beneath each scenario, there were some facial expressions represented as emotional story outcomes. There were ten main components of TEC as follows.

In the *External* understanding level, there were three elements: *recognition*, *situation*, and *reminders*. *Recognition* mainly tested children’s understanding of facial expressions, such as *happy*, including naming and recalling (e.g., *What do you call these facial expressions?*); *Situation* primarily tested how children understand emotions in social context (e.g., *How did he/she feel when he/she got the gift from Mom?*); *Reminders* mainly examined how children interpret the emotions of protagonists based on the clues provided (e.g., *He/she was happy before*, *how did he/she feel when seeing dog’s death?*). The total scores of *External* level ranged 0 to 24.

In the *Internal* understanding level, three elements were: *desires*, *belief*, and *display*. *Desires* were primarily about understanding emotions when the protagonist’s wishes were fulfilled or unfulfilled (e.g., *How did he and she feel after being given an apple?*); *Belief* examined participants put themselves in other’s position to understand emotions (e.g., *How did he/she feel before opening the toy box?*); *Display* tested children’s understanding of emotional exposure within the constraints of social interaction rules (e.g., *How did he/she feel as the winner while his/her friend was the loser?*). The total scores of *Internal* level ranged 0 to 16.

In the *Reflective* understanding level, four components were: *mixed*, *regulation*, *morality*, and *causes*. *Mixed* was about understanding the complex and diverse emotions of the same protagonist (e.g., *How did he/she feel when falling off the bike and being laughed at by others?*); *Regulation* mainly tested how participants understand the ability of protagonists to control and regulate their emotional state (e.g., *Can he/she would feel good though his/her cat was dead?*); *Morality* was supposed to analyze how the sample understands emotions in a moral context (e.g., *How did he/she feel after stealing candy of someone else?*), *Causes* would examine how children interpret the reasons behind emotions (e.g., *Why was he/she so angry?*) [54]. The total scores of *Reflective* level ranged 0 to 37. The total scores of TEC was 77. The higher the score, the stronger the comprehension.

In order to avoid the sequential effect, a Latin-square design was used to establish the sequence of all facial expressions and combined emotional scenario stories. Each session lasted approximately 5–10 min.

### 2.4. Data Analysis

The data were collected from ten tasks designed to measure children’s understanding of emotions. All the data were analyzed using SPSS 25.0. According to the systematic procedures of the existing research [12,13,14,15,55], these ten elements were divided into three levels (*External*, *Internal*, *Reflective*) for analysis. The MANOVA was conducted to examine whether there would be differences in the three levels of understanding emotions, including differences between gender and children’s status. Besides, the comparison of LBC cared for by different caregivers was carried out to further analyze the impact of changes in actual caregivers and split family.

## 3. Results

The MANOVA on three levels of EU was performed. The result of *External* emotions understanding level showed that gender had no main effect (*F*_(3, 174)_ = 1.97, *p* = 0.12, *η*^2^ = 0.03) and no significant interaction with children’s status (*F*_(3, 174)_ = 2.30, *p* = 0.09, *η*^2^ = 0.04). The main effect of children’s status was significant (*F*_(3, 174)_ = 11.34, *p* = 0.00 < 0.001, *η*^2^ = 0.16). Further analysis revealed that *recognition* (*F*_(1, 179)_ = 13.50, *p* = 0.00 < 0.001, *η*^2^ = 0.07), *situation* (*F*_(1, 179)_ = 20.79, *p* = 0.00 < 0.001, *η*^2^ = 0.11), and *reminders* (*F*_(1, 179)_ = 7.80, *p* = 0.006 < 0.01, *η*^2^ = 0.04) also showed a significant difference. NLBC scored significantly higher than the LBC.

The results of *Internal* emotion understanding level indicated that there was also no main effect on gender (*F*_(3, 174)_ = 3.75, *p* = 0.12, *η*^2^ = 0.06). Moreover, the interaction between gender and children’s status was not significant as well (*F*_(3, 174)_ = 1.01, *p* = 0.38, *η*^2^ = 0.02). On the contrary, children’s status showed the main effect (*F*_(3, 174)_ = 7.21, *p* = 0.00 < 0.001, *η*^2^ = 0.11). The main effect of children’s status was reflected in the *desires* (*F*_(1, 179)_ = 18.90, *p* = 0.00 < 0.001, *η*^2^ = 0.10) and *display* (*F*_(1, 179)_ = 9.35, *p* = 0.003 < 0.01, *η*^2^ = 0.05), instead of *belief* (*F*_(1, 179)_ = 1.63, *p* = 0.20, *η*^2^ = 0.01). NLBC scored significantly higher than the LBC on the components as *desires* and *display*.

Furthermore, the results of *Reflective* showed that the main effect of gender (*F*_(4, 173)_ = 3.02, *p* = 0.02 < 0.05, *η*^2^ = 0.07) and children’s status (*F*_(4, 173)_ = 4.19, *p* = 0.003 < 0.01, *η*^2^ = 0.09) were both significant. However, the interaction effect between gender and children’s status was not significant (*F*_(4, 173)_ = 0.18, *p* = 0.95, *η*^2^ = 0.004). In terms of four components of *Reflective*, gender has a main effect on *morality* (*F*_(1, 179)_ = 4.47, *p* = 0.04 < 0.05, *η*^2^ = 0.03). Girls scored significantly higher for understanding moral emotions than boys. Other elements including *mixed* (*F*_(1, 179)_ = 0.47, *p* = 0.50, *η*^2^ = 0.003), *regulation* (*F*_(1, 179)_ = 2.52, *p* = 0.11, *η*^2^ = 0.01) and *causes* (*F*_(1, 179)_ = 3.17, *p* = 0.08, *η*^2^ = 0.02) did not present main effect. The main effect of children’s status was shown in the components as *mixed* (*F*_(1, 179)_ = 4.60, *p* = 0.03 < 0.05, *η*^2^ = 0.03) and *causes* (*F*_(1, 179)_ = 16.32, *p* = 0.00 < 0.001, *η*^2^ = 0.09). LBC scored significantly higher than NLBC on the understanding of *mixed* and *causes* (see Table 1). There was no main effect of the children’s status in the comprehension of *regulation* (*F*_(1, 179)_ = 0.63, *p* = 0.43, *η*^2^ = 0.004) and *morality* (*F*_(1, 179)_ = 0.15, *p* = 0.70, *η*^2^ = 0.001) (see Table 1).

Since the main differences were examined between LBC and NLBC from the three levels of EU, the *t*-test of the total scores of understanding emotions was conducted to further explore whether there are differences in the ability of LBC and NLBC to understand emotions as a whole. The result indicated that the total score of NLBC was significantly higher than that of LBC (*t*_(178)_ = 5.34, *p =* 0.00 < 0.001, *Cohen’s d =* 0.80). This implied that NLBC actually had much higher levels of understanding emotions than LBC at five to six years of age.

One-way ANOVA of total scores of LBC’s emotion understanding between different caregiver types, e.g., one parent, grandparents, or other relatives, was performed. The results indicated that the differences between the three groups did not reach statistical significance (*F*_(2, 89)_ = 0.82, *p =* 0.44, *η*^2^ *=* 0.01).

## 4. Discussion

In rural China, long-term parental migration in search of better jobs in the cities was a fact of life. LBC should deserve more research attention, even children in their preschool period. This study investigated the three developmental levels of emotional understanding competence between five- and six-year-old LBC and NLBC. The results showed that the experience of parental absence was truly correlated with the LBC’s weaker EU, regardless of level. Further analysis revealed that there was no significant change in EU among LBC being taken care of by one parent, grandparents, or other relatives.

### 4.1. Poor Emotional Understanding of LBC

The present study demonstrated that the EU competence of rural LBC aged five to six years was significantly lower than that of NLBC at any of the three levels (*External*, *Internal*, *Reflective*). This was closely consistent with the previous studies and conclusions [42,56]. Emotional development fluctuated intricately in the preschool years. Young children begin to experience sophisticated emotional events in real life. Between the ages of two and four, children may pair these kinds of familiar situations with appropriate emotional reactions, and they may come to understand which desire affected which kinds of emotions at five to six years old [57,58]. In this way, it was easy for them to assess what makes individuals’ emotional reactions so different. Children’s three levels of EU competence could benefit from training and social setting factors as potential moderators [59]. However, longer duration of being left behind was significantly associated with more total difficulties in psychological adjustment and emotional cost [1,60]. Specifically, LBC fell behind in EU regarding facial expressions, situations, reminders, desires, display rules, morality, external causes, and mixed features. On the whole, the early emotional comprehension ability of LBC was indeed adversely affected by the left-behind experience.

### 4.2. Family Emotional Function Would Make Sense in LBC’s EU

This study also confirmed that once the family structure changed or became unstable because of parental migration. No matter who acted as actual caregiver of the LBC, the impact on emotional understanding ability showed basically no difference. Family system theory argues that parental presence is irreplaceable for the development of infants and toddlers [35]. With regard to emotional faces, for example, a previous study indicated that the facial expressions of parents, such as mothers, could bring about significant fluctuations in children’s affective reactions, regulatory coping behaviours, and meaning-making [61]. However, LBC had poor emotional interactions about emotions with their parents and primary caregivers. For example, migrant parents usually hide their emotions to prevent their children from missing them. Besides, not being a permanent adoption, actual guardians might not show their own true emotions to these LBC in some cases. Adverse family life experiences in early childhood would create a disadvantage in emotional comprehension, such as facial emotion understanding [62,63]. Even if only one parent left home, another parent could not make up for the emotional lack young children experience [64]. In other words, once the operation of the family emotional function was affected due to parental migration, the LBC would have a similar disadvantage in understanding emotions regardless of who the actual caregivers are.

### 4.3. Left-Behind Experience Effected Early EU Left-Behind

Chinese LBC with attachment problems fare less well, both physically and emotionally, compared to Chinese children growing up in stable homes [65]. Particularly in early childhood, the quality of attachment with parents was correlated with the social-emotional development of children [66]. For example, a previous study indicated that children’s emotion regulation had close associations with attachment security [67]. During preschool, the ability to process emotions would actually depend on the level of attachment security [68]. It is difficult for young LBC at home to have access to such psychological resources. From the inter-group comparison of LBC, it was found that a split family might bring negative psychological effects and sensitivity to safety issues.

Children’s differences in early emotional understanding were related to specific aspects of family experiences [69]. This early adverse deprivation of emotional experience may have a negative impact on the recognition of facial emotion [70]. The results showed that LBC at five and six-years old fall behind in processing and identifying some facial expressions like sad and fearful. A previous study of LBC with urban Internet-addiction showed that they focused more on negative emotions [71]. The younger the age of separation, the greater the negative effects on children’s emotional cognition and experience [72,73]. It is clear that children who are left behind in early childhood are more affected by parental absence and perform less well in emotional understanding.

### 4.4. Limitations

The present study had several limitations that should be recognized. The emotional comprehension of LBC may be affected by a variety of factors, especially for preschool children at a critical stage of social development. For instance, the length of separation needs to be further verified in the future. Besides, there was no statistical difference of EU in the types of caregivers of LBC, which may be due to the relatively small number of participants who were recruited by a non-randomized sampling method in Guangdong province in southeast China. Caution must be taken when generalizing the reported findings to LBC from other communities.

## 5. Conclusions

The present study investigated the three levels (*External*, *Internal*, *Reflective*) of emotional understanding of LBC and NLBC. Indeed, the emotional understanding competence of young LBC was significantly worse than that of NLBC at any level. Overall, LBC’s early emotional understanding competence were at a disadvantage. Among the three types of caregivers, the early internal differences of LBC were not significant, even when one of parents did not migrate. Emotion understanding is crucial for early social interaction and adjustment. Therefore, this was an important reference for exploring the development of emotional understanding in younger and larger age groups. To reduce the effect of negative psychological suffering of LBC in early childhood, efforts should be made to prevent the occurrence of being left behind, so that parents can shoulder the responsibility of parenting, and all can obtain support.

## Figures and Tables

**Table 1:** Comparison of EU between two groups of children.

		Children’s Status					
		LBC (n = 90)		NLBC (n = 90)		*F*	95% CI
		M	SD	M	SD		
External	recognition	7.98	3.44	9.77	3.10	13.50 ***	[−2.75, −0.83]
	situation	2.71	0.14	3.52	0.10	20.79 ***	[−1.16, −0.46]
	reminders	1.83	1.49	2.40	1.42	7.80 **	[−0.97, −1.66]
Internal	desires	3.44	0.21	4.68	0.19	18.90 ***	[−1.79, −0.67]
	belief	0.88	0.13	1.13	0.16	1.63	[−0.66, 0.14]
	display	1.34	0.11	1.74	0.08	9.35 **	[−0.65, −0.14]
Reflective	mixed	2.74	0.17	3.27	0.17	4.60 *	[−1.00, −0.04]
	regulation	1.17	0.07	1.10	0.05	0.63	[−0.10, 0.23]
	causes	13.08	0.73	16.53	0.46	16.32 ***	[−5.14, −1.77]
	morality	0.44	0.10	0.39	0.10	0.15	[−0.23, 0.34]

Note: * *p* < 0.05. ** *p* < 0.01. *** *p* < 0.001. Abbreviations: LBC, left-behind children; NLBC, non-left-behind children.

## Data Availability

The data that support the findings of this study are available from the corresponding author upon reasonable request.
